# A Step towards Achieving Sustainable Otologic Surgery in Low-Resource Settings: A Cost Comparison between Shipping an Otologic versus Microscopic Surgical Setup

**DOI:** 10.3390/audiolres12040039

**Published:** 2022-07-13

**Authors:** Rachel Thompson, Gregory Basura, Taseer Feroze Din, Asitha Jayawardena

**Affiliations:** 1 University of Minnesota Medical School, Minneapolis, MN 55455, USA; thom4795@umn.edu; 2 Department of Otolaryngology, University of Michigan, Ann Arbor, MI 48109, USA; gbasura@med.umich.edu; 3 Department of Pediatric Otolaryngology, Stanford University, Stanford, CA 94305, USA; tdin@stanford.edu; 4 Children’s Minnesota, Pediatric ENT and Facial Plastics, University of Minnesota, Minneapolis, MN 55455, USA

**Keywords:** ear surgery, global surgery, endoscopic otologic surgery, cost effectiveness

## Abstract

**Background:** The advancement of otologic surgery in low-resource settings has been limited by the cost and transport of surgical equipment. This study compared the transportation costs of an otologic microscopic surgical setup (MSS) versus an endoscopic surgical setup (ESS) in low- and low to middle-income countries (LMICs) for surgical teaching. **Methods:** Dimensions of microscopes, endoscopes and associated surgical instruments were used to calculate shipping costs from Minneapolis, MN, USA to Kenya, Haiti and Sri Lanka. **Results:** The average cost of internationally shipping the ESS is less than the MSS in Kenya (ESS: USD 1344.03; MSS: USD 20,947.00; *p* = 0.370), Haiti (ESS: USD 549.11; MSS: USD 1679.00; *p* < 0.05) and Sri Lanka (ESS: USD 945.38; MSS: USD 8490.57; *p* = 0.377). Freight shipping was required for the MSS while the ESS can be packed into an international checked bag for USD 35.00 USD. **Discussion:** The ESS has fewer logistical barriers than the MSS, making the endoscope a feasible option for surgical teaching in LMICs.

## 1. Introduction

Over 430 million people globally are affected by disabling hearing loss, including 34 million children [1]. The Global Burden of Disease Study in 2019 reported 1.57 billion people living with at least a 20 decibels or more hearing loss [2]. The economic burden associated with hearing loss exceeded USD981 billion globally in 2019 with 57% of these costs coming from non-high-resource countries [3].

In children, conductive hearing loss is prevalent (>90%) due to otitis media with effusion, tympanic membrane perforation or cholesteatoma [4]. There is a higher burden of hearing loss due to environmental factors and infectious etiologies in low- and low to middle-income countries (LMICs) which often require surgical management for definitive treatment, unlike genetic causes of sensorineural hearing loss that are identified more frequently in high resource countries [4,5]. Accessible surgical equipment is an obstacle to the surgical management of these conditions in LMICs [6].

Surgical techniques in otology continue to evolve and endoscopic approaches to the middle ear are increasingly implemented. Endoscopic approaches have shown improved anatomic visualization [7]. with similar audiometric and surgical outcomes seen with binocular approaches including tympanic membrane graft success rate [8], operative time, and cholesteatoma recidivism [9,10,11]. Furthermore, endoscopic techniques lead to a mean cost reduction compared to the traditional microscopic otologic surgical approaches [12].

Despite the high prevalence and societal burden of untreated hearing loss, otologic surgery is often a lower priority for otolaryngologists in LMICs as other acute pathologies often take precedence. To this end, various teaching missions have sought to teach otologic surgery in LMICs to local otolaryngologists [13,14]. A major barrier to teaching otologic surgery in these countries is the cost and maintenance of otologic surgical equipment. The endoscopic approach has been successfully used as a teaching tool in Malawi to train midlevel providers in endoscopic myringoplasty to increase the accessibility of the procedure [15]. and the endoscope has been discussed as a tool with a shorter learning curve than microscopic ear surgery [7].

Understanding the cost of shipping equipment is important for sustainability as many LMICs do not have the ability to repair surgical equipment locally. Though major capital cities in Africa have access to microscopes, these are not easily transported outside of these cities or internationally. Here, we evaluated the cost of establishing an otologic teaching program in LMICs and specifically the shipping costs for endoscopic and microscopic surgical setups.

## 2. Methods

The dimensions and weight of endoscopes, microscopes and instrument sets were obtained from vendors in the United States. Standard Storz otoendoscopes were used for dimension data for the endoscopic approach. The Zeiss OPMI Pico was selected as a representative microscope given its broad availability, cost effectiveness, small footprint, and ability to connect to any external monitor for teaching purposes. As any external monitor can be used to connect to the HDMI port of the microscope, the dimensions of the microscopic monitor were not included in the setup cost for the microscope as this equipment can be purchased locally. However, the monitor for the endoscope is proprietary equipment that must be purchased through Storz and therefore was included in the dimensions of the endoscopic setup. 

Shipping costs were determined based on quotes received from UPS, DHL and FedEx regarding microscopic and endoscopic shipping. Rates were obtained from Minneapolis, MN to Port-au-Prince, Haiti, Nairobi, Kenya and Colombo, Sri Lanka. Maximum weight for international shipping to these countries is 32 kg (70 pounds) based on international regulations, therefore heavier item shipping requires freight shipping [16]. Freight and standard shipping costs were determined from a drop off by the sender and direct delivery to the recipient hospitals. Customs fees and insurance fees were not included in the cost analysis. Prices were compared by independent student *t*-tests to determine statistical significance.

Baggage prices were determined for international flights leaving from Minneapolis, MN to Port-au-Prince, Haiti, Nairobi, Kenya and Colombo, Sri Lanka. A standard checked bag can weigh up to 22.5 kg for USD 35. An overweight bag can weigh up to 45 kg with 22.5 to 33 kg bags at a price of USD 200 and bags of 33 to 45 kg at a price of USD 400 per bag. Carry-on baggage must be 22 cm × 35 cm × 56 cm and is free on international flights.

## 3. Results

The combined weight of the endoscopic setup (scopes, monitor, container, surgical equipment) was 16.74 kg. The combined weight of the microscopic setup (microscope, surgical equipment) was 95.4 kg. The average cost of shipping the endoscopic surgical set up was significantly less when shipping to Haiti (endoscopic cost: USD 549.11, microscopic cost: USD 1679.00; *p* < 0.05). Shipping the endoscopic surgical setup was also found to be less expensive than the microscopic setup when shipping to Nairobi, Kenya (endoscopic cost: USD 1344.03, microscopic cost: USD 20,947.00; *p* = 0.370) and Colombo, Sri Lanka (endoscopic cost: USD 945.38, microscopic cost: USD 8490.57; *p* = 0.377), however, this was not statistically significant secondary to a greater variation in shipping cost (Table 1). For the vendor DHL, the cost of shipping the endoscopic surgical set up was more expensive than the microscopic surgical set up to Colombo, Sri Lanka (endoscopic cost: USD 798.99; microscopic cost: USD 693.00) when including the endoscopic monitor.

Furthermore, the endoscopic surgical setup can fit into an international checked bag for USD 35.00 USD (in addition to the cost of airfare) given that a standard bag can weigh up to 22 kg. The Zeiss OPMI PICO microscope, even if disassembled, could potentially be placed in two “very overweight” bags (33 to 45 kg) at a price of USD 400/bag and a total of USD 800 (in addition to the cost of airfare). The surgical tools themselves, regardless of the endoscopic or microscopic setup, could be placed in a standard carry-on bag for no additional costs (Table 2). 

## 4. Discussion

For an international trip from the US, endoscopic otologic setup costs are cheaper than binocular microscopic equipment. Freight shipping, the only means to transport a microscope due to the large size and weight, has various limitations which may make it not clinically feasible to mobilize this set up in LMICs. Freight shipping requires a business to place the order, which may make it difficult for individual surgeons to organize. Freight shipping can also be more variable in cost depending on the shipper and location, as FedEx shipping, due to the specific weight, size and shape of the microscope, was notably higher in cost than other companies to ship a microscopic setup to Kenya (USD 59,795). In comparison, the endoscopic surgical setup can easily fit into a checked bag for a nominal fee, which is desirable over dismantling and then reassembling a microscope for transport in multiple overweight checked bags. Given this variability, less emphasis was placed on the statistical significance, or lack of significance, of these results and rather the real-life implications of a several thousand dollar difference in shipping costs. In a low-resourced region with a limited surgical budget, particularly where the surgical capital itself may be donated and only the shipping costs are borne by the local surgeons, this difference in shipping may be the difference between obtaining or not obtaining a specific surgical setup.

Although international surgical teaching work has been limited in the COVID-19 pandemic, [17] eliminating the surgical burden of ear disease remains an important aspect of medical outreach initiatives throughout the world. As such, the endoscope should be considered due to its ease of use, and lower transportation cost, making it logistically more feasible to repair if necessary. Additionally, the endoscope is a versatile surgical tool that can be used to increase a country’s ability to surgically treat ear disease, and to treat both rhinologic [18] and laryngologic pathology [19]. This may be particularly useful in rural settings that do not have microscopes or access to local or international repair services. 

This study is limited in that it relies on vendor data regarding dimensions for each piece of equipment. Shipping pricing varies between companies and countries and is constantly changing based on global market demand. Consistency in freight shipping (air) was challenging to obtain given restrictions within individual countries. These data do not account for maintenance costs, an important and necessary factor in international work. We anticipate maintenance costs to be similar for both microscope and endoscope; however,, because the microscope is transported via crate shipping, it is vulnerable to additional transport and damage costs compared to endoscopes which can be transported via hand luggage.

Lastly, the concept described in this paper is not feasible for all surgeons. Surgical familiarity with the endoscope and mastoid drilling using surgical loupes is necessary for the techniques described in this paper to be effective. Ideally, both microscope and endoscopic surgical setups would be utilized, as is the case in high resource countries. However, due to cost limitations, if a surgeon in an LMIC must choose one setup, the data in this paper favors the endoscope as a cost-effective means of primary surgery. Future research, which may be more feasible upon the widespread return of international surgical travel, could evaluate the cost differences between the endoscope and microscope in actual practice settings and could examine the impacts of maintenance, facility fees and costs offset by donations.

Although it may seem intuitive that a larger microscope may be more expensive to ship than an endoscope, this paper is the first to provide quantitative data highlighting this difference. Therefore, the data in this paper can help support surgical teams in operative planning and budgeting when it comes to creating a sustainable otologic surgical program in a low-resource setting.

## 5. Conclusions

The endoscope has less variability in shipping price compared to the microscope, and when shipping to certain low-resourced areas of the globe, may be a more cost-effective surgical setup than the microscope.

## Figures and Tables

**Table 1:** International Shipping Fees: All fees are from packages dropped off in Minneapolis and directly delivered to international hospitals without accounting for insurance or customs fees. All microscope shipping fees are for air freight shipping.

	Minneapolis, MN to Nairobi, Kenya (USD)	Minneapolis, MN to Port-au-Prince, Haiti (USD)	Minneapolis, MN to Colombo, Sri Lanka (USD)
UPS			
Endoscope	1354.59	580.38	967.12
Microscope	1519.49	1417.66	1066.08
FedEx			
Endoscope	1361.76	581.12	1070.03
Microscope	59,795.00	1940.33	23,712.63
DHL			
Endoscope	1315.74	485.82	798.99
Microscope	1529.40	N/A	693.00
Average Price			
Endoscope	1344.03	549.11	945.38
Microscope	20,947.96	1679.00	8490.57
Significance	0.370	**0.011**	0.377

**Table 2:** Baggage Fees: Fees are determined based on United States airline pricing when flying internationally from the United States. The endoscopic set up will fit in one standard bag at USD 35.00 per bag. The microscope will fit into two “very” overweight bags at USD 400.00 per bag with the remaining surgical tools going in a carry-on bag.

	Dimensions (cm)	Weight (kg)	Estimated Checked Baggage Cost (USD)
**Endoscope**			
Scope			$35
Storz 0°	0.3 × 14	2.27	
Storz 30°	0.3 × 14		
Container system (up to 4 scopes)	46.0 × 27.9 × 10.2		
Monitor, camera	63.5 × 63.5 × 20.32	9.07	
Surgical Tools			Free (carry-on)
Storz Endoscopic Ear Set	59.7 × 27.9 × 20.3	5.4	
Total Weight		16.74	
**Microscope**			
Scope			
Zeiss OPMI Pico	65 × 142.5 × 173	90	$800
Surgical Tools			Free (carry-on)
Storz Middle Ear Set	59.7 × 27.9 × 20.3	5.4	
Total Weight		95.4	
